# CD123‐targeting immunotherapeutic approaches in acute myeloid leukaemia

**DOI:** 10.1111/bjh.70019

**Published:** 2025-07-24

**Authors:** Alexandra Dreyzin, Noa G. Holtzman, Challice L. Bonifant

**Affiliations:** ^1^ Center for Cellular Engineering National Institutes of Health Clinical Center Bethesda Maryland USA; ^2^ National Cancer Institute National Institutes of Health Bethesda Maryland USA; ^3^ Division of Transplantation and Cellular Therapy, Sylvester Comprehensive Cancer Center University of Miami Miller School of Medicine Miami Florida USA; ^4^ Department of Pediatrics Johns Hopkins University School of Medicine Baltimore Maryland USA; ^5^ Department of Oncology, The Sidney Kimmel Comprehensive Cancer Center Johns Hopkins University School of Medicine Baltimore Maryland USA

**Keywords:** acute leukaemia, antibody therapy, cellular engineering, cellular therapies, immunotherapy, myeloid leukaemia antigens

## Abstract

Treatment of relapsed or refractory acute myeloid leukaemia (AML) has remained a significant challenge, with limited available targeted immunotherapies. CD123, or the interleukin‐3 (IL‐3) receptor, has long been known as a potential target expressed on AML blasts, and a range of different approaches to targeting CD123 have been trialled with variable anti‐tumour responses and toxicities observed. Here, we review the clinical outcomes of these therapies, including monoclonal antibodies and antibody‐drug conjugates, as well as bispecific T‐cell engager molecules and chimeric antigen receptor (CAR) T cells. We discuss the potential reasons for therapy‐associated toxicity and limited efficacy, including differential antigen expression, the AML microenvironment and patient‐derived T‐cell fitness. To address these challenges, we highlight novel approaches to CD123 targeting currently in preclinical development. Promising new strategies include combination therapies that target CD123 and other known AML‐associated antigens such as CD33 or CLL‐1, NK‐cell‐based cell therapies, and bispecific‐secreting T cells.

## CD123 AS AN AML‐ASSOCIATED THERAPEUTIC TARGET

Identifying immunotherapy targets for acute myeloid leukaemia (AML) has been challenging due to disease heterogeneity and overlap in surface antigen expression between AML blasts and normal haematopoietic cells. CD123 is the alpha chain of the interleukin‐3 receptor (IL‐3Rα). This antigen is overexpressed on the leukaemia surface relative to that on normal myeloid cells, with enriched high expression on immature leukaemia‐initiating cells (LIC).^1^, ^2^, ^3^ Binding of IL‐3 to its receptor activates the JAK/STAT, MAPK and PI3K pathways to promote myeloid differentiation. Antibodies against CD123 are cytotoxic to myeloid leukaemia stem cells in vitro and have anti‐tumour activity in AML mouse models.^3^, ^4^

The direct relationship of CD123 to leukaemogenesis is not clear, but in AML patients, CD123 expression is associated with higher disease burden, increased blast proliferation and poor prognosis in both adults and children.^5^, ^6^, ^7^ Higher CD123 expression has been observed in association with high‐risk molecular prognostic markers, including the *NUP98:NSD1* gene rearrangement, *NPM1* mutation and *FLT3* activation.^8^, ^9^, ^10^, ^11^ In samples from patients before and after relapse, CD123 expression is variable, with the relapse likely comprised of a subclonal expansion of populations with more or less CD123 expression.^8^ Interestingly, in patients with Fanconi anaemia, CD123 overexpression on haematopoietic stem cells is associated with AML development.^12^

CD123 expression can also be identified in other malignancies that variably show positivity, namely, blastic plasmacytoid dendritic cell neoplasm (BPDCN), myeloproliferative neoplasms, myelodysplastic syndrome, B‐cell acute lymphoblastic leukaemia (ALL), hairy cell leukaemia, chronic lymphocytic leukaemia (CLL) and transformed B‐cell chronic lymphoproliferative disease.^2^, ^13^, ^14^ Furthermore, the level of CD123 expression at diagnosis in both AML and B‐ALL is predictive of persistent measurable residual disease (MRD) after induction therapy.^15^ Given its consistent expression and association with disease progression and relative chemoresistance, CD123 is thus an attractive immunotherapeutic target.

### Antigen‐specific toxicity considerations

Prior to the clinical translation of CD123 targeted therapies, potential toxicities were considered and studied in animal models. Expected toxicities are those secondary either to generalized inflammation or specific to ‘on‐target, off‐tumour’ binding of CD123 by the tested immunotherapeutic. CD123 is expressed on differentiated myeloid cells, haematopoietic stem and progenitor cells and activated T, B and natural killer (NK) cells with potential direct cytotoxicity within the haematopoietic compartment.^16^, ^17^, ^18^ CD123 expression is also evident on endothelial cells, with this expression increasing in the presence of inflammatory cytokines.^19^, ^20^ The pattern of CD123 tissue expression has led to concern and vigilance for capillary leak in safety studies. Because non‐human primates (NHPs) have CD123 tissue distribution comparable to that of humans, the safety and tolerability of CD123 targeting was initially tested in cynomolgus monkeys. A variety of immunotherapies were trialled in these animals, including antibodies, antibody‐drug conjugates (ADCs) and bispecific engager molecules. Encouragingly, NHPs treated with the CD123 monoclonal antibody talacotuzumab had no sign of generalized inflammatory toxicity, but the therapy caused peripheral basophil, dendritic cell and NK‐cell depletion while sparing pluripotent progenitor cells.^21^, ^22^ Testing of the bispecific T‐cell engaging molecule XmAb14045 (vibecotamab) did not result in systemic toxicity, but was also associated with haematopoietic cell alterations, with CD4 and CD8 margination observed along with persistent marrow depletion of CD123+ cells.^23^ Similarly, treatment of NHPs with a different bispecific CD123xCD3 T‐cell engager, flotetuzumab, was associated with transient leucopenia, neutropenia and thrombocytopenia, with no observed effect on haematopoietic marrow progenitors.^23^, ^24^ A large NHP study (*n* = 32) of serially dosed flotetuzumab reported an association with cytokine release with a ‘first‐dose effect’—elevation of IL‐6 occurred hours after the first infusion, but was decreased following subsequent, even higher doses.^4^ In contrast to the above studies, infusion of the diphtheria toxin/anti‐IL3R drug conjugate DT388IL3 led to high rates of malaise and anorexia, severe alanine aminotransferase (ALT) elevation in 2/6 treated monkeys and development of a lethal vasculitis in one animal given a high dose of the drug.^25^, ^26^ Taken together, NHP testing of a range of CD123‐targeted immunotherapies was not associated with severe inflammatory toxicities but did cause haematopoietic cell depletion. In these studies, myeloid stem and progenitor cells were not affected. Although one animal treated with a relatively high dose of a toxic agent developed a lethal vasculitis, capillary leak syndrome was not observed otherwise. The tolerability observed in non‐human primates justified translation to human studies.

## CLINICAL EXPERIENCE WITH CD123 TARGETING

**FIGURE 1:**
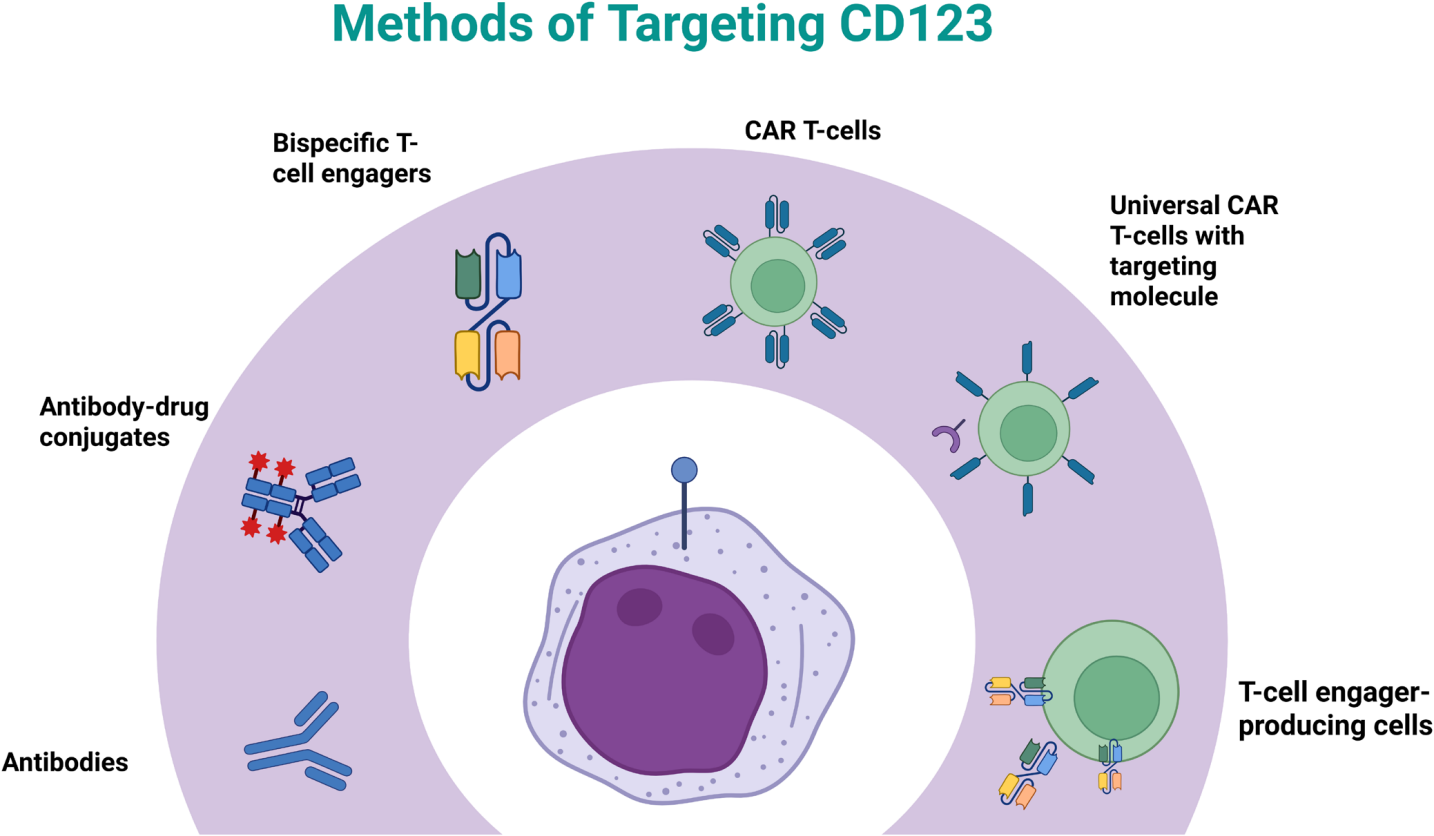
Overview of CD123 targeting methods. Immunotherapeutic targeting strategies range from monoclonal antibodies, to drug‐conjugates, to cellular therapies such as CAR T cells, with an increasing repertoire of modified or augmented cellular therapies.

**FIGURE 2:**
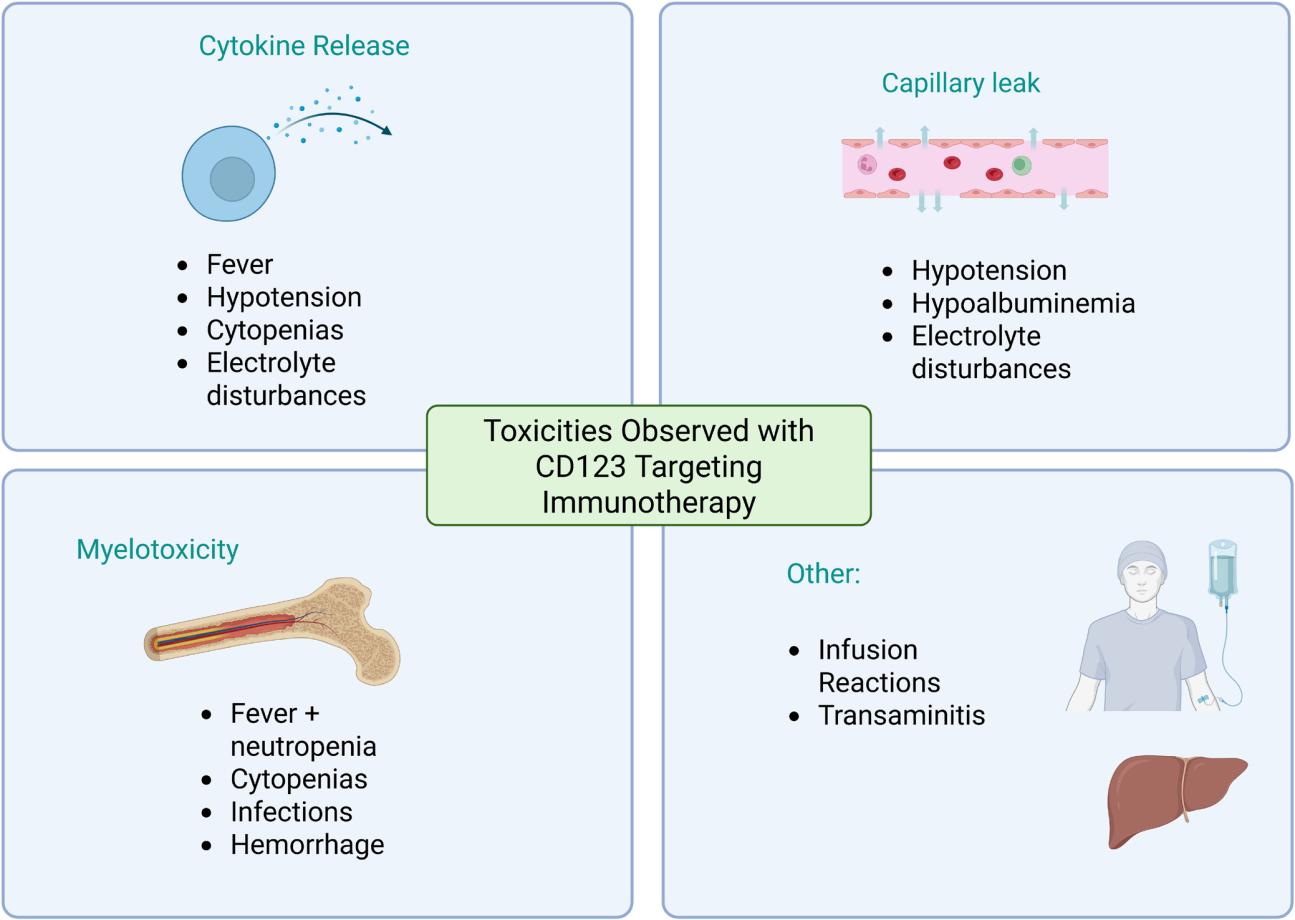
Toxicities observed with CD123‐targeting immunotherapy. The most common toxicities observed were related to cytokine release or myelosuppression, although capillary leak, infusion reactions and elevated transaminases have also been observed.

Several CD123 targeting immunotherapies have now been developed and tested in clinical studies in humans, including antibodies, ADCs and cellular therapies (Figure 1). Below, and in Tables 1 and 2, we describe published anti‐AML CD123‐targeting data available to date. Figure 2 further illustrates the reported toxicities observed in clinical trials thus far.

**TABLE 1:** CD123 targeting antibody therapies.

Agent	Mechanism of action	Phase, NCT#	Combination therapy	No. of AML patients	Adverse events/DLTs	Efficacy	References
Talacotuzumab (CSL362)	Humanized IgG1 monoclonal antibody	2/3, NCT02472145	Decitabine	167	84% Grade 3 AEs, 33% leading to death: sepsis (4.8%), MOF (5.4%), pneumonia (3.4%), septic shock (3.4%), sudden death (0.7%)	15% CR	Montesinos et al.^27^
Tagraxofusp (SL401)	ADC	1/2, NCT02270463		40	Grade 3 TRAEs: ALT elevation (15%), AST elevation (15%); CLS (5%)	2.5% CR; 5% PR, 10% blast reduction	Frankel et al.^28^
Tagraxofusp	ADC	1b, NCT03113643	Azacitidine ± Venetoclax	56	Grade ≥3 AEs: febrile neutropenia (29%), anaemia (21%), infection (14%), ALT elevation (7%), TLS (7%), CLS (5%)	For Tag‐VEN‐AZA group: 39% CR, 19% Cri, 12% MLFS For Tag‐AZA: 7% CRi	Lane et al.^29^
IMGN632 (Pivekimab)	ADC	1/2, NCT03386513		91	Grade 3 AEs: febrile neutropenia (10%), infusion reaction (7%) and anaemia (7%). 1 treatment‐related death.	21% ORR, 17% CR at Phase 2 dose	Daver et al.^30^
IMGN632 (Pivekimab)	ADC	1, NCT04086264	Azacitidine + venetoclax	61	Grade 3 TEAEs in >20%: febrile neutropenia (30%), infusion reaction (21%)	31% CR, 51% ORR	Daver et al.^31^
Flotetuzumab	DART	1/2, NCT02152956		88	Grade ≥3 in 8%; 3 DLTS: delirium, confusional state, Gr 3 CRS; CRS in 81%	CR 11.7%, ORR 24%; ORR 30% at Phase 2 dose	Uy et al.^32^
APVO436	BiTE	1b, NCT03647800		46	Grade 3 AEs: IRR in (28%), CRS (21%), transient neurotoxicity (11%)	6% CR, 65% SD	Uckun et al.^33^
Vibecotamab (XmAb14045)	BiTE	1, NCT02730312		118	TEAEs in 87.5%; anaemia (25.8%), febrile neutropenia (25%), pneumonia (27.5%). Gr 5 TEAE in 8 pts. (6.7%), including pneumonia, sepsis, cerebral haemorrhage, CRS, pulmonary oedema; DLT: 13.3%, including CRS, infusion reaction, GGT increase and hypertension.	9% ORR (11.5% among those who received target dose); 48% SD	Ravandi et al.^34^
Vibecotamab (XmAb14045)	BiTE	2, NCT05285813		18	Grade 1–3 Infusion reactions in 68%, minimal myelosuppression.	28% MRD negativity (among patients with morphologic remission with +MRD)	Nguyen et al.^35^
JNJ‐63709178	BiTE	1, NCT02715011		62	Grade ≥3 TEAEs in 83.9%: CRS (43.5%), Alt increase (19.4%), AST increase (14.5%), fever (2.9%), infusion reactions (11.3%). DLTs: 2 (3.2%) Grade 5 CRS, Grade 3 infusion reaction	No CR	Boyiadzis et al.^36^

Abbreviations: ADC, antibody–drug conjugate; AE, adverse event; BiTE, Bispecific T‐cell Engager; CLS, capillary leak syndrome; CR, complete response; CRi, complete response with incomplete count recovery; CRS, cytokine release syndrome; DART, dual‐affinity re‐targeting protein; DLT, dose‐limiting toxicity; MOF, multiple organ failure; MRD, minimal residual disease; ORR, overall response rate; PR, partial response; SD, stable disease; TEAE, treatment‐emergent adverse event; TLS, tumour lysis syndrome; TRAE, treatment‐related adverse event.

**TABLE 2:** CD123 targeting CAR T‐cell therapies.

CAR product	Sponsor	Phase, NCT#	Vector	Dose	LD	No. of patients	Paediatric or adult	CRS	DLTs	Efficacy	References
RNA CART123	University of Pennsylvania	1, NCT02623582	Electro‐porated mRNA	4e6 × 3–6 doses	Optional Cy	7 enrolled, 5 treated	Adult	80% w/CRS; 58% with Gr3+	No DLTs	No disease response observed	Cummins et al.^37^
Autologous CD123CAR T‐cells	City of Hope Mustang Bio	1, NCT02159495	Lentiviral	50–500 M	Flu/cy	31 enrolled, outcomes from 7 reported (6 AML)	Adult	71% with Grade 1–2 CRS	no DLTs	1 CR (2 months), 1 CR to BMT, 1 sustained CR, 2 with reduced blasts but no CR	Budde et al.^38^
Autologous CD123CAR T‐cells	University of Pennsylvania	1, NCT03766126	Lentiviral	1–5 × 10^6^ CAR cells/kg	Flu/cy	20 enrolled, 12 treated	Adult	83% with CRS; 50% with Grade ≥3	2 DLTs: Grade 5 CRS	2/12 achieved CR and 1 CRi	Bhagwat et al.^39^
Autologous CD123CAR T‐cells	University of Pennsylvania	1, NCT04678336	Lentiviral	2e6–1e7 CART123 cells/kg	Flu/cy		Paediatric (to age 25)				Egan et al.^40^
Autologous CD123CAR T‐cells (CATCHAML)	St. Jude's Research Hospital	1, NCT04318678	Lentiviral	3e5–1e7 cells/kg	Flu/Cy	12 enrolled, 5 treated	CAYA	100% Gr 1 CRS vs. isolated fevers	No DLTs	1 CR, 1 PR	Naik et al.^41^
UniCAR02‐T‐CD123	Technical University Dresden	1, NCT04230265	Lentiviral	Up to 500 M, with up to 4 mg/day TM	Flu/cy	19 treated	Adult	63% with Gr1‐2 CRS, 1 with Gr 3 CRS, 1 with neurotoxicity	1 DLT: hypofibrinoginaemia	8/15 ORR; 3/4 ORR in MRD population. 1 pt. reported CR for 5 months, 1 with reduction in MRD able to go to transplant.	Ehninger et al.^42^
UCART123/AMELI‐01 (allogeneic CD123 CAR T cells)	Cellectis Weill Cornell MDACC	1, NCT03190278	Lentiviral and TALEN	1.25e5 to 3.3e6 cells/kg	Flu/Cy +/− alemtu‐zumab	18 enrolled, 17 treated	Adult	100% with CRS, 4 with Grade ≥3, 2 with HLH, 2 with ICANS	4 DLTs: CRS Gr 3–5	4/16 with evidence of activity; 1 MRDneg CR, 2 SD, 1 MLFS	Sallman et al.^43^

Abbreviations: BMT, bone marrow transplant; CR, complete response; CRi, complete response with incomplete count recovery; CRS, cytokine release syndrome; cy, cyclophosphamide; DLT, dose‐limiting toxicity; Flu, fludarabine; HLH, haemophagocytic lymphohistiocytosis; ICANs, immune‐effector cell associated neurotoxicity; MLFS, morphologic leukaemia‐free state; MRD, minimal residual disease; ORR, overall response rate; PR, partial response; SD, stable disease.

### CD123‐targeting antibodies and ADCs

CD123 antibodies alone have had limited efficacy in patients. Talacotuzumab (JNJ‐56022473, CSL‐362), a humanized, IgG1 monoclonal CD123 antibody, was tested in a cohort of patients with refractory AML who could not tolerate chemotherapy and/or who were hypomethylating agent (HMA) refractory.^44^ Alone, antibody administration led to toxicity (infusion reactions and infections) without notable disease response. A second trial of talacotuzumab for patients in morphological remission who were at high risk of relapse was more promising, with 10 of 20 treated patients having a sustained CR at 6 months.^45^ The combination of talacotuzumab with decitabine was also tested in a similar population of patients not eligible for standard chemotherapy. Ultimately, no improved rate of complete remissions was observed, and the therapy was associated with cytopenias, infusion reactions and febrile neutropenia.^27^ Encouragingly, while antibody treatment was ultimately insufficient, talacotuzumab therapy was associated with greater reduction of CD123+ bulk disease than decitabine alone, suggesting targeted activity.

The combination of antibody‐based binding and cytotoxic payload delivery is often more effective than treatment with an antibody alone. It follows that agents linking CD123 targeting with directed cytotoxicity have been more effective. The antibody‐drug conjugate tagraxofusp is the human IL‐3 cytokine linked to a diphtheria toxin. To test this agent, 45 patients with refractory AML were treated in a Phase I trial; of these, one patient achieved a complete response (CR) and two had a partial response (PR).^28^ Toxicities associated with the therapy included capillary leak syndrome, fever, transaminitis and hypocalcaemia. Tagraxofusp delivery has thus been shown safe and can be effective in patients with AML. A trial extending monotherapy to the combination of tagraxofusp and azacitidine or venetoclax was recently completed. Treated patients were either newly diagnosed and ineligible for chemotherapy or with relapsed/refractory (R/R) disease.^29^ Following dose escalation, 26 patients were treated at the identified expansion dose (12 μg/kg daily for 3 days). The overall response rate to combined therapy was 69%, with the majority of responders achieving MRD negativity. High rates of capillary leak syndrome (47%) were also reported.^29^ A Phase II trial of this treatment regimen is ongoing (NCT06456463). The combination of tagraxofusp and a second ADC that binds CD33, gemtuzumab ozogamicin, is also being tested as a therapy for paediatric and adult patients with AML (NCT05716009). It is notable that while capillary leak was not observed in prior animal studies of CD123 targeting, the complication has been frequently seen in clinical trials of tagraxofusp. Similarly, when tagraxofusp was tested as treatment for BPDCN, ~20% of patients were diagnosed with CLS.^46^ According to retrospective FDA analysis of all patients treated with tagraxofusp, up to 55% of patients met criteria for capillary leak, and 2% died due to complications of this syndrome.^47^

Another antibody‐drug conjugate, pivekimab sunirine (IMGN632), is a CD123 antibody linked to a DNA‐alkylating drug. IMGN632 first demonstrated efficacy in vitro against CD123‐expressing AML and ALL cell lines^48^, ^49^ and given this promise, 91 patients with R/R AML were treated on a Phase 1/2 trial (NCT03386513). At the identified target dose (0.045 mg/kg every 3 weeks), 17% achieved a CR. Toxicity was minimal, with <10% of patients experiencing infusion reactions and cytopenias; however, one treatment‐related death of unknown cause was reported.^30^ There is an ongoing Phase I/II trial of IMGN632 combined with venetoclax and azacitidine (NCT04086264) for patients with R/R AML. Thus far, reported data include 61 treated patients, with 51% achieving an objective response and a CR rate of 31%.^31^ Capillary leak was not reported, and toxicity was minimal. It is encouraging that neither NHP anti‐CD123 targeting studies, monoclonal antibody therapy, nor IMGN632 treatment were associated with CLS. Even so, capillary leak has not generally been observed in patients receiving diphtheria toxin‐coupled therapies such as tagraxofusp. Given that this toxicity has been observed more consistently in the context of endothelial cell‐expressed targets like CD123 or IL‐2, it is concerning (but not definitive) that CD123 targeting rather than the drug conjugate is the primary determinant of this toxicity.^50^, ^51^

### Dual targeted antibodies

Bispecific engager molecules are immunotherapies that are engineered with dual targeting capability, designed to bring together two discrete cell types. For example, linking a single chain variable fragment (scFv) binding an AML‐associated surface antigen to an scFv that binds a component of the T‐cell receptor will link effector T cells to AML blasts and promote cytotoxicity via T‐cell receptor clustering and activation. Flotetuzumab is such an engager, as a dual affinity retargeting antibody (DART) containing an scFv that binds CD123 and a second that binds CD3ε.^52^ Flotetuzumab was first tested as salvage therapy for patients with R/R AML and in this context, treatment achieved a response rate of 30% (NCT02152956).^32^ Interestingly, while a specific association between TP53 mutations and CD123 expression has not been reported, patients with disease that carried TP53 mutations were more likely to respond to flotetuzumab, likely due to a favourable disease environment for immune‐mediated anti‐leukaemia activity.^53^

APVO436 is another bispecific antibody, composed of a humanized CD3 and fully human CD123 binding domain, linked to a human IgG1 Fc.^33^ It was tested in a Phase I trial in patients with R/R AML (NCT03647800), in which the dose was escalated from 0.3 to 60 μg weekly. Infusion reactions and cytokine release syndrome were observed, but these were neither dose‐limiting toxicities nor associated with disease burden, even at the highest dose.^33^ Encouragingly, of 34 patients with AML, 2 patients achieved first a PR, followed by a CR at over 90 days from infusion. In addition, 22 treated patients achieved disease stability at best response, with eight patients sustaining this stability for over 3 months.

A third CD123xCD3 small molecule engager, vibecotamab (XmAb14045), was tested in a phase 1 trial of 120 patients with R/R AML (NCT02730312).^34^ The majority of treated patients (59.2%) experienced mild (grade ≤2) cytokine release syndrome (CRS) indicating drug activity, but only 9% of patients achieved a CR. Notably, all responders had a baseline marrow blast count of <25%. Dose limiting toxicity occurred in 16 (13%) patients, and one patient experienced Grade 5 CRS. There were an additional seven patients who died due to treatment‐emergent adverse events, including infection, cerebral haemorrhage and pulmonary oedema. In this study, higher vibecotamab dosing was associated with greater CRS incidence and severity. The study team did not report an association between CRS and disease burden; however, the presence of severe CRS in a small subset of patients suggests the need to stratify treatment and to identify biomarkers that predispose patients to inflammatory toxicity. In recently reported results of a Phase II study of vibecotamab, 28% of patients with MRD+ AML achieved MRD negativity, with no CRS reported, possibly due to low baseline disease burden at the time of treatment.^35^

Finally, JNJ‐63709178 is another CD123xCD3 targeting dual antibody for which a Phase I trial (NCT02715011) has been completed, including 62 adult patients with AML.^36^ While the majority of treated patients experienced adverse events including CRS, fever, infusion reactions or transaminitis, there were minimal disease responses even at the highest tested doses.

Extending the concept of dual targeting, a CD3 engager molecule built on a DARPin (Designed ankyrin repeat protein) scaffold that allows for specific binding to multiple targets has been engineered to bind CD33, CD123 and CD70. This agent is currently being tested in a European clinical trial (NCT05673057).^54^ This molecule also has an extended half‐life as compared to other engaging molecules and can be dosed weekly after initial step‐up dosing. To date, results from 37 enrolled patients are available, with 30 patients evaluable for response after treatment.^55^ Of these, one achieved CR and three achieved a morphologic leukaemia‐free state as defined by study investigators, with 50% of patients having evidence of overall blast reduction. The majority of patients (65%) experienced CRS, but no dose‐limiting toxicities were observed.

In summary, T‐cell engaging small molecule agents targeting AML have shown some efficacy, but inflammatory toxicities have been more common than disease response. Bispecific antibodies that engage NK cells rather than T cells may have the potential for preserved anti‐tumour efficacy with less cytokine release.^56^ NK cells express CD16, the FcγR, which serves to activate antibody‐dependent cellular cytotoxicity (ADCC) when antibody‐bound cells are then linked to NK cells via the Fc portion of the antibody. A CD123XCD16A engager has therefore been tested in a Phase I clinical trial in patients with relapsed/refractory AML.^57^ Among 24 treated patients, four achieved a CR, including three of six patients treated at the highest dose level (300 mg once weekly). CRS occurred in two patients and resolved without additional intervention, while fatal pneumonitis was observed and was dose‐limiting in one patient. With additional pharmacologic engineering, a trispecific CD123‐NK cell engager (TriKE) binding CD123, CD16A and the NK cell activating receptor NKp46 was trialled in 42 patients with AML and a 12% remission rate was observed.^58^ In this trial, there were no dose‐limiting toxicities, and cytokine release was rare, with Grade 1 CRS occurring only in two patients. A combination trial testing the TriKE together with venetoclax and azacitidine is currently ongoing (NCT06508489).

A significant disadvantage of scFv‐based small molecule engager therapies is their short half‐life. Because of rapid metabolism and clearance, small molecule engagers are typically administered via continuous infusion. Innovations in engager engineering include incorporation of an Fc domain to engender increased stability; however, these larger molecules have diminished efficacy likely due to inability to facilitate formation of an effective cellular synapse and risk additional toxicities due to their potential for complement activation.^59^ Even so, the incorporation of an Fc domain has resulted in improved pharmacokinetics for CD3 engagers targeting CD19 and CD20, and a CD123xCD3 engager molecule that incorporates an Fc domain is being readied for Phase I trials.^60^, ^61^, ^62^ Aside from the pharmacokinetic limitations, bi‐ and trispecific engager molecules depend on the fitness of the engaged effector cell population to facilitate directed cytotoxicity. In AML patients, T and NK cells are inherently dysfunctional, limiting the therapeutic efficacy of engager therapy.^63^, ^64^ These immunological effector cells are also suppressed by the systemic chemotherapy administered as standard of care for disease control.

### CAR T‐cell therapies

While T‐cell engager molecules tend to be short‐lived with efficacy dependent on the fitness of patients' effector cells, chimeric antigen receptor (CAR) T cells can provide a proliferating reservoir of activated, targeted effector cells. CAR T‐cell therapy is a rapidly growing therapeutic class in which T cells are engineered to express an HLA‐independent synthetic antigen‐binding receptor linked to activation and costimulatory domains and used for adoptive transfer to patients. A number of CD123‐targeted CAR T‐cell products have been developed with both academic and industry sponsorship. Single‐target autologous CD123 CAR T cells are currently in trials as treatment for paediatric and adult patients. Although much of the data from these studies are only available in abstract form, these provide important insights into the potential toxicity and efficacy of the therapy. The first reported use of CD123 CAR T cells was described in a case report of a single patient with AML refractory to several lines of therapy who relapsed after allogeneic BMT and ultimately received CD123‐targeted CAR T cells.^65^ The cell therapy infusion led to CRS that was responsive to the IL6‐receptor blocking antibody, tocilizumab. The patient had minimal disease response, with bone marrow blast reduction observed from 59% to 45%. A second abstract reporting the use of CAR T‐cell therapy tested in seven patients targeting CD123 described mRNA electroporation as a manufacturing method, with the resultant T‐cell product demonstrating transient CD123‐CAR expression.^37^ There were no detectable CAR+ cells found in the circulation of any patient post‐infusion and no anti‐leukaemia activity observed, likely due to the choice of a non‐integrating cell modification in a rapidly proliferative T‐cell product.

The first evidence of anti‐AML activity of CD123 CAR T cells was observed in a dose‐escalation study of a CD123 CAR T‐cell product carried out at City of Hope: two of six treated patients achieved a CR, one at the starting dose level of 5 × 10^7^ CAR T cells and one at dose level 2 at 2 × 10^8^ CAR T cells.^38^ Subsequent data from a study at the University of Pennsylvania testing CD123 CAR T cells manufactured with viral CAR integration reported a clinical response in 25% of 12 treated adult patients with AML. Notably, the majority (10/12) of treated patients experienced CRS with two Grade 5 events reported. CRS incidence was not correlated with baseline disease burden. Correlative laboratory data suggested that the high levels of cytokines defining CRS may actually be inhibitory to AML blast apoptosis.^39^ CD123‐CAR T‐cell trials are not restricted to adult patients; two ongoing trials are evaluating CD123‐CAR T‐cell therapies in paediatric patients (NCT04318678—St Jude, NCT04678336—Children's Hospital of Philadelphia).^40^ An initial abstract from the first five patients treated in the St. Jude study reported achievement of CRs in two patients and blast reduction in a third.^66^ The manufacturing procedure for this study was subsequently modified to include dasatinib in the ex vivo CAR T‐cell culture with the aim of preserving T‐cell stemness and improving efficacy^67^; however, this manufacturing change was associated with increased toxicity. Among six patients whose cells were exposed to dasatinib, all experienced CRS, including three with severe toxicity (Grade 4–5 CRS).^41^ Results from the Children's Hospital of Philadelphia study have not yet been reported.

Typically, CAR T cells are manufactured using a therapy‐specific vector for genetic modification. In an attempt to decrease manufacturing costs, Bachmann et al.^68^ developed UniCAR T cells to express an adaptor CAR originating from a universal vector sequence with an extracellular domain designed to bind to infusible linkers that can then be engineered to bind a variety of antigens, including CD123. The efficacy of this platform is ultimately dependent not only on CAR T‐cell potency and trafficking but also on the interaction between CAR T cells and targeting molecules. Of 10 patients who completed treatment with an autologous UniCAR targeting CD123, all experienced CRS, two achieved a CR and four achieved a PR.^69^ A dose‐finding trial of a related, allogeneic product, Allo‐RevCAR01‐T cells with CD123 targeting molecules, is ongoing (NCT05949125).^42^

Because of concerns about autologous, patient‐derived, CAR T‐cell fitness and the challenge of controlling AML progression during the time needed for CAR T‐cell manufacture, healthy donor‐derived CAR T cells have also been tested. UCART123 cells are a product in which transcription activator‐like effector nuclease (TALEN) mediated genetic disruption of CD52 and the T‐cell receptor was used to mitigate the associated risks of rejection and graft versus host disease.^43^ UCART123 has been tested in a Phase I trial in which reported toxicities were similar to those seen with autologous CAR T cells, including CRS in 15/16 patients, with Grade 4 CRS observed in two patients and Grade 5 CRS in one. Despite this high toxicity rate, disease responses were modest. Of the 16 patients treated, one had a CR, one had a PR and two had stable disease.^43^ A case report of another donor‐derived CD123 CAR T‐cell product described the use of the CAR T‐cell therapy as part of a conditioning regimen prior to stem cell transplant from the same haplo‐identical donor.^70^ The treated patient had evidence of CAR T‐cell expansion, Grade 4 CRS and reduction of blasts prior to his transplant, but unfortunately died of complications secondary to acute graft‐versus‐host disease (GVHD) after transplant.

It is worth noting that there are a number of early phase trials of CD123‐targeting CAR T cells recruiting patients outside the United States, although limited details regarding product or patient characteristics, trial design and/or clinical outcomes are currently available (NCT04272125, NCT06420063, NCT06006403, NCT06125652 and NCT05995041).

## POTENTIAL MECHANISMS OF POOR RESPONSE TO CD123‐TARGETED IMMUNOTHERAPY

**FIGURE 3:**
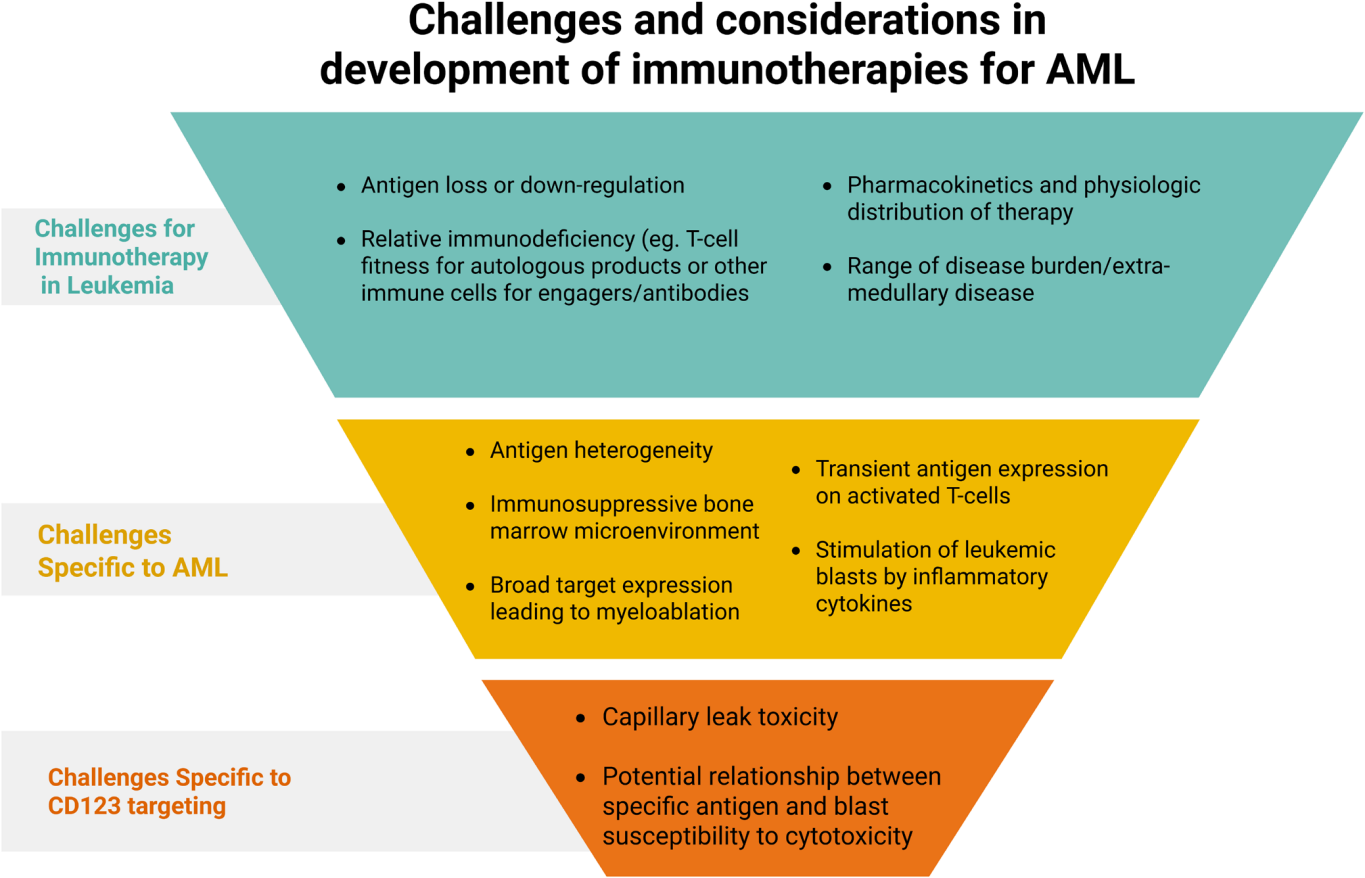
Challenges and considerations in development of immunotherapies for AML. Many of the known mechanisms of resistance to immunotherapy apply broadly across targets and disease types, while some challenges are particular to AML treatment. More research is needed to elucidate whether there are target‐specific mechanisms that alter immunotherapy response. AML, acute myeloid leukaemia.

Limited anti‐AML responses following CD123‐targeting immunotherapy are likely due to a number of factors including disease heterogeneity with variable CD123 expression, interference and immunosuppression within the disease microenvironment and poor patient T‐cell fitness. These challenges are broadly shared by immunotherapies generally and specifically by AML‐targeted agents as depicted in Figure 3.

Antigen heterogeneity has been a powerful barrier to precise AML immunotherapeutic targeting. In a real‐world cohort, cells expressing CD123 comprised 5%–75% of patient samples.^15^ In one large study, CD123 was reportedly more commonly expressed at initial diagnoses than at the time of relapse, when immunotherapy is more likely to be introduced.^8^ Given this clear variability, it is not surprising that, among the CD123‐targeting trials described above, disease response has been partial at best, with some incomplete reduction in CD123+ blasts observed. Exposure to a CD123‐targeting agent has the potential to induce antigen downregulation or selective depletion of antigen‐positive malignant cell populations. Loss of CD123‐positive cells in residual disease has been observed after tagraxofusp treatment in patients with BPDCN.^71^, ^72^

The immune microenvironment plays a crucial role in the innate response to malignancy and profoundly impacts immunotherapeutic function. It is striking that immune features of bone marrow samples from patients with AML can prognosticate responses to chemo‐ and immunotherapy.^53^ AML cells secrete inhibitory cytokines (TGFβ, IL‐10, IL‐35) and limit the secretion of T‐cell activators (IL‐18, IFNγ).^73^, ^74^ The cytokine profile of patients with AML differs from that of patients with ALL in the setting of CRS.^75^ Not likely unique to CD123‐targeting, data from a recent trial of CD123 CAR T cells highlight the possibility of these inflammatory cytokines promoting AML blast survival.^39^ AML blasts directly inhibit T‐cell activation via the expression of ligands such as PD‐L1 and galectin‐9, which bind the T‐cell immunosuppressive checkpoint receptors PD‐1 and TIM‐3 and are upregulated by exposure to IFNγ.^76^ This phenomenon was observed in patients who were treated with a CD33‐targeting bispecific engager molecule,^77^ and it is expected but has not yet been reported for CD123‐targeting agents. AML blasts can also trigger metabolic changes that inhibit T‐cell function. For example, the presence of AML blasts can lead to adipocyte lysis and fatty acid release, which is inhibitory to T‐cell function.^78^ On a larger scale, malignancy‐induced vascular changes in the bone marrow limit perfusion and prevent oxygen, nutrients, drugs and cellular therapies from reaching the blast microenvironment.^79^ The hypoxia induced by vascular remodelling may also limit T‐cell function and proliferative capacity within the bone marrow.

Like other immunotherapies, many CD123‐targeting therapies are either composed of T cells or rely on engagement of a patient's own T cells. Suppressive T‐cell populations, such as regulatory T cells (Tregs), exhausted T cells or those that are terminally differentiated and senescent, are less likely to contribute to an anti‐tumour response. To wit, when higher proportions of Tregs or cells with exhaustion markers were identified in final CAR T‐products, treated patients were less likely to have a clinical response.^80^ Importantly, patients treated with higher proportions of naïve or central memory cells in their CAR T‐cell products were more likely to have a clinical response.^81^, ^82^ At baseline, patients with AML have lower proportions of naïve T cells at diagnosis compared with healthy patients or even those with ALL.^83^ Paediatric patients with AML at diagnosis or exposed to chemotherapy similarly had a lower proportion of T cells fit for CAR T manufacture than those with ALL.^84^ Furthermore, among children, the innate T‐cell response to AML has been found to differ from the response to ALL: in a recent study, T cells from children with ALL, AML or mixed‐phenotype acute leukaemia (MPAL) were compared, and only those from patients with ALL had leukaemia‐specific responses.^85^ Interestingly, when stimulated with IFNγ, cells from AML patients were able to recover cytotoxicity against AML blasts in vitro. Exposure to cytarabine, which is a major component of induction chemotherapy for AML, is specifically associated with T‐cell exhaustion and differentiation, which may explain why cells from patients with AML are less likely to produce functional CAR T cells compared with those of ALL patients^86^ The more advanced age of typical AML patients may also contribute to T‐cell exhaustion.^87^ Unique to CAR T‐cell therapies, CD123 is upregulated on proliferating T cells, which may lead to CD123 CAR T‐cell fratricide.^16^, ^20^ While it has not been directly reported in the context of CD123, trogocytosis, the process by which CAR T cells bind and express surface markers from target cells, may also exacerbate fratricide.

While there are general challenges to cell and immunotherapeutic efficacy that are shared regardless of disease target, there are also target‐specific mechanisms of disease resistance. The CD19 and CD22 targeting experiences have shown that immunotherapeutic direction towards different antigens can result in variable efficacy and unique toxicity even when antigen expression densities are similar.^88^, ^89^ Interestingly, a recent trial demonstrated the response of CD19+ AML to CD19‐targeting CAR T cells, highlighting the concept that CAR T‐cell binding of the right target antigen may generate sufficient T‐cell stimulation to overcome disease‐related barriers to CAR T‐cell expansion and anti‐tumour efficacy.^90^ Further comparative studies or multi‐targeted approaches are needed to fully relate AML‐directed CAR T‐cell resistance to target antigen choice.

## EMERGING CD123‐TARGETING THERAPIES

**FIGURE 4:**
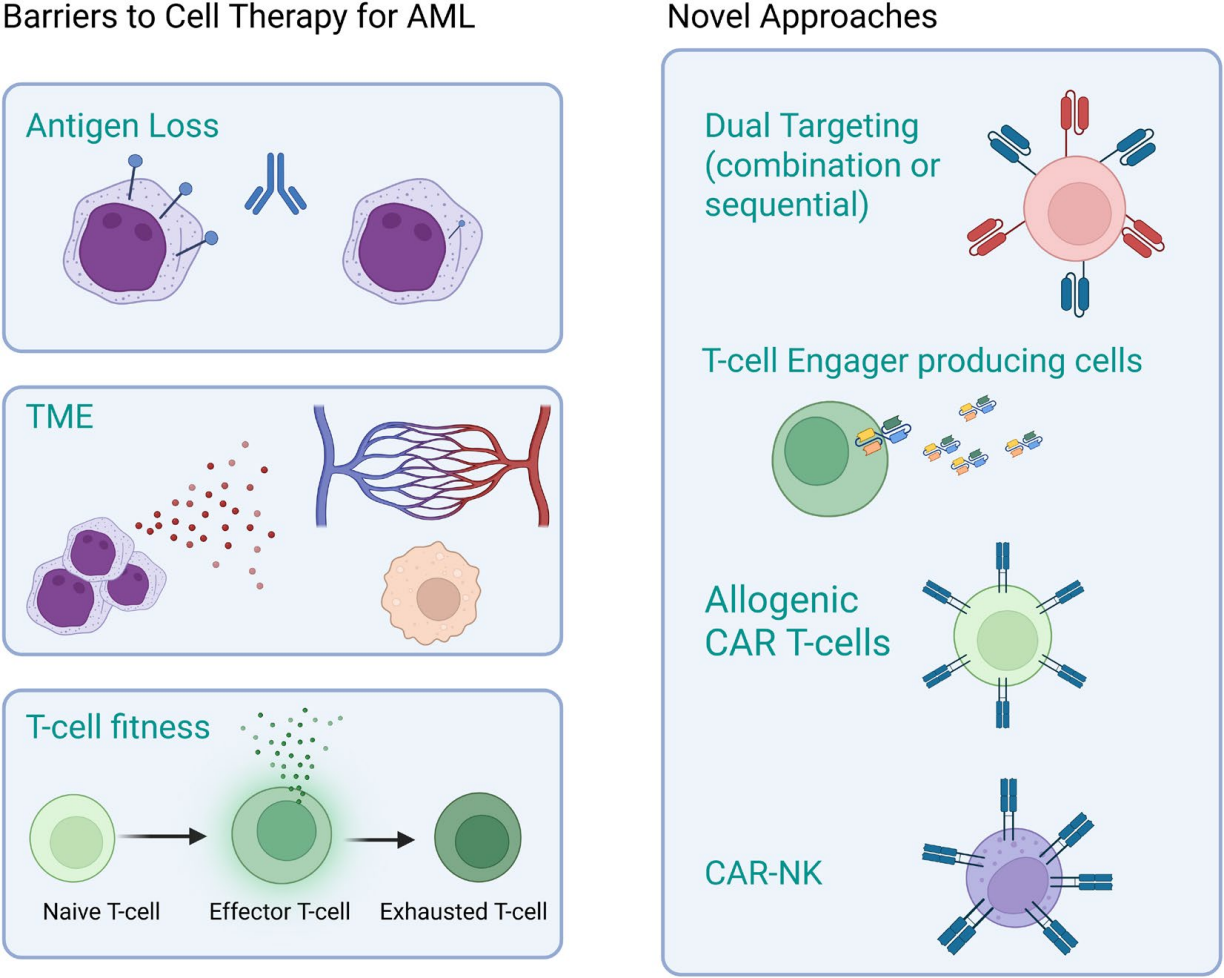
Barriers to AML Immunotherapy and novel CD123 targeting approaches. A number of different hypotheses have been proposed to explain poor efficacy of immunotherapy and cellular therapies for AML. Specific to CD123 targeting are antigen loss or heterogeneity, as well as the risk of off‐tumour toxicity. Broadly, immunotherapy is hindered by the immunosuppressive tumour microenvironment (TME) and baseline T‐cell dysregulation in patients with AML. Dual targeting, whether combined in one therapy or given sequentially, may help overcome antigen heterogeneity. Cellular therapies with enhanced cytotoxicity, such as T cells that secrete cytokines or engager molecules in vivo may help to overcome immune suppression. Finally, use of alternative cell types such as allogenic T cells or NK cells may provide an alternative to suboptimal autologous T cells. AML, acute myeloid leukaemia.

Novel CD123‐targeting immunotherapies that may address some of these observed barriers to optimal anti‐AML activity are in preclinical development. Promising strategies include the incorporation of multiple‐antigen targeting, combination of pharmacologic and cellular therapies and the use of non‐patient derived cells, such as allogeneic T cells or NK cells as effectors (Figure 4).

Dual‐antigen targeting may compensate for antigen loss, inconsistent CD123 expression and disease heterogeneity. A dual‐targeted CD123 and CD33 CAR T cell has successfully eradicated AML in xenograft models.^91^ To increase specificity, the co‐stimulatory and activating domains of the CAR molecules are split between scFvs, so that both CD33 and CD123 are necessary on the target cell for full T‐cell activation. Another example of a bispecific CAR T cell that has shown in vitro cytotoxicity combines targeting of CD123 with that of the folate receptor β, which is expressed on about 70% of AML blasts.^92^ An alternate strategy includes modifying a CAR T cell to target the NKG2D ligand to recruit NK cells while activating T‐cell cytotoxicity via the expressed CAR.^93^ Sequential targeting of different antigens also has potential. This could be accomplished by sequential infusion of different CAR T cells or with the use of adaptor CAR T cells that are activated when combined with an adaptor molecule specific to the target of interest.^94^ The use of unique adaptor molecules binding different targets with short half‐lives potentially allows for an ‘off‐switch’ as a safety feature.

A novel category of cellular therapies is T cells that secrete bispecific antibodies. These combine bystander T‐cell activation derived from bispecific engager molecule binding with the proliferative capacity and powerful effector function of ex vivo activated and expanded T cells. As a cell‐based therapy, these are more durable than infusion of bispecific small molecules alone and allow for persistent activity and concentrated secretion of the dual‐binding agents at targeted tumour sites. First described as brain tumour therapy, we subsequently developed a CD123xCD3 engager molecule that can be expressed and constitutively secreted by T cells as a direct stimulant of cytotoxicity against CD123+ AML.^95^, ^96^ While CAR T cells with CD28 or 41BB co‐stimulatory domains and an identical CD123‐binding scFv develop tonic signalling and an ‘exhaustion’ immunophenotype during manufacture, CD123xCD3 T‐cell engager‐secreting T cells (ENG‐T) retain a phenotype most similar to unmodified T cells despite activation and ex vivo expansion.^97^ Because of the observed severe cytokine release syndrome associated with CD123‐targeted engagers and CAR T cells, any therapy that aims to increase cytotoxic activity must also consider safety. To this end, our CD123xCD3 ENG‐T also incorporates a CD20 ‘off‐switch’, which can be activated by infusion of the FDA‐approved antibody, rituximab. Additional modifications can be added to ENG‐T cells to enhance activity, such as expression of inducible co‐stimulatory molecules or cytokines, such as IL‐15.^98^, ^99^ A Phase I clinical trial is currently in development at the National Cancer Institute (NCI) designed to test a CD123xCD3 ENG‐T‐cell product in adult patients with R/R AML.

Finally, NK and other effector cell types are an attractive alternative to T cells, especially in a patient population with autologous T cells likely to have diminished fitness. CAR NK cells have been manufactured from healthy donors and designed to target CD123. These have demonstrated efficacy in mouse models with minimal associated toxicity.^100^, ^101^ In an attempt to improve function and persistence, CD123‐CAR NK cells have been engineered to secrete IL‐15; however, while this improved the in vitro activation and effector cell functionality, it also led to lethal toxicity.^102^

In summary, CD123 targeting as a component of AML immunotherapy has thus far been limited by inadequate anti‐tumour cytotoxicity despite clear evidence of cell activity in vitro. This suggests a need to overcome AML and microenvironmental inhibition as well as to improve effector cell functional persistence. Correlative immunophenotypic studies evaluating changes in both T cells and blasts after CD123‐targeting therapy will be important for further elucidating mechanisms of resistance. As anti‐tumour activity is intentionally strengthened, careful monitoring for intensified inflammatory and on‐target, off‐cancer toxicities with consideration of abortive treatment will be critical. While a majority of patients in CD123‐targeting immunotherapy trials have experienced mild CRS, a subset has had severe associated morbidity and mortality, highlighting the need to identify risk factors for toxicity prior to immunotherapy.

As advances in CD123 targeting and in general, to immunotherapies, are made, an important question will be where these modalities fit in the context of current standard of care chemotherapy regimens. Analogous to the CD33‐targeted gemtuzumab, antibody‐drug conjugates, such as tagraxofusp, may be more easily integrated into upfront chemotherapy, depending on CD123 expression at diagnosis. Immunotherapy is not likely to replace chemotherapy, but these agents are useful adjuncts to be used in combination or as second‐line therapies in the setting of relapse. Given concern for toxicity related to disease burden, chemotherapy could also be used for initial disease debulking, with immunotherapy then implemented to eliminate low‐level disease prior to consolidative haematopoietic cell transplantation. Ultimately, targeting of CD123 for AML treatment will most likely achieve optimal success when used as a component of a multi‐agent combinatorial treatment plan.

## AUTHOR CONTRIBUTIONS

A.D. contributed to conceptualization and writing—review and editing. N.G.H. contributed to conceptualization, supervision and writing—review and editing. C.L.B. contributed to conceptualization, supervision and writing—review and editing.

## FUNDING INFORMATION

This research was funded in part by the Alex's Lemonade Stand Foundation, the American Cancer Society and the St. Baldrick's Foundation for Childhood Cancer. N.G.H. is a K12 Scholar supported through Award Number K12CA226330 from the National Cancer Institute of the National Institutes of Health. Figures created using Biorender.

## CONFLICT OF INTEREST STATEMENT

C.L.B. has been awarded and has pending patent applications describing the use of engineered T and NK cells as therapeutics. C.L.B. has received research support from Bristol‐Myers Squibb.

## DISCLAIMER STATEMENT

The views, information, or content, and conclusions presented do not necessarily represent the official position or policy of, nor should any official endorsement be inferred on the part of, the Clinical Center, the National Institutes of Health, or the Department of Health and Human Services.

## Data Availability

All references are cited for this review paper. It does not contain original data.
